# Supplementary material for: The evolution of the natural killer complex; a comparison between mammals using new high-quality genome assemblies and targeted annotation
Source: Immunogenetics. 2017 Feb 9;69(4):255–69. doi: 10.1007/s00251-017-0973-y (PMC5350243; doi:10.1007/s00251-017-0973-y)
Supplement: Supplementary file 1 — (DOCX 359 kb) [file 251_2017_973_MOESM1_ESM.docx]

**Supplementary Material**

The evolution of the natural killer complex; a comparison between mammals using new high-quality genome assemblies and targeted annotation

John C. Schwartz^1^, Mark S. Gibson^1*^, Dorothea Heimeier^1^, Sergey Koren^2^, Adam M. Phillippy^2^, Derek M. Bickhart^3^, Timothy P. L. Smith^4^, Juan F. Medrano^5^, and John A. Hammond^1†^

^1^ Livestock Viral Diseases Programme, The Pirbright Institute, Ash Road, Pirbright, Surrey GU24 0NF, UK

^2^ National Human Genome Research Institute, Bethesda, MD 21702, USA

^3^ Animal Genomics and Improvement Laboratory, USDA-ARS, Beltsville, MD 20705, USA

^4^ Meat Animal Research Center, USDA-ARS, Clay Center, NE 68933, USA

^5^ Department of Animal Science, University of California, Davis, Davis, CA 95616, USA

^*^ Current: CEDOC, Faculdade de Ciências Médicas, Universidade Nova de Lisboa, 1150-082 Lisbon, Portugal

^†^ Corresponding author

Phone: (+44)1483231397

E-mail: [john.hammond@pirbright.ac.uk](mailto:john.hammond@pirbright.ac.uk)


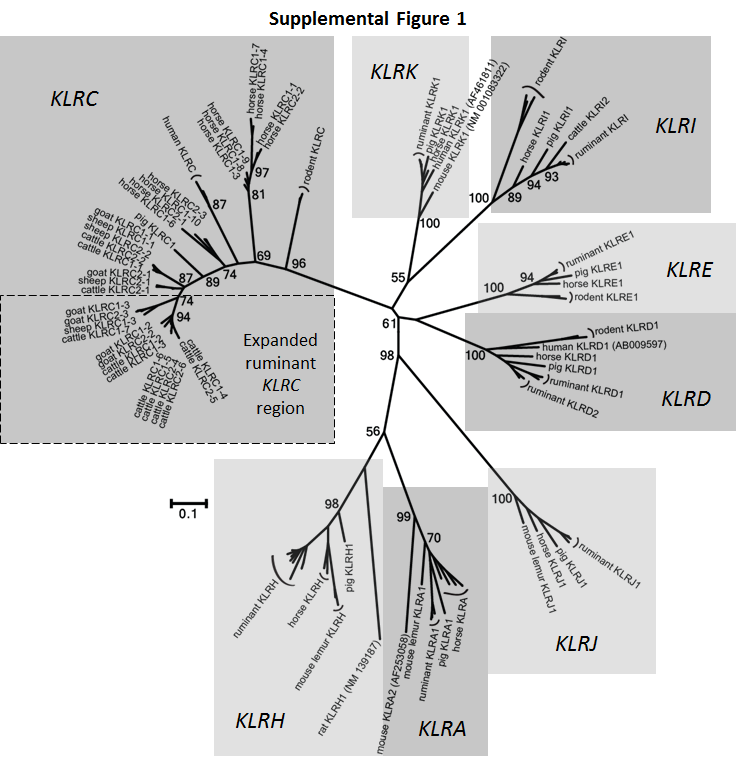


**Supplementary Fig. 1** Phylogenetic relationships of nucleotide coding region sequence for 3’ lectin domain exons of NKC genes in cattle, goats, sheep, pigs, horses, and selected genes from humans, lemurs, rats, and mice. In all cases, “ruminant” refers to cattle, sheep, and goat. Bootstrap values (out of 100) are indicated at branch points. Sequence accessions for the ruminants, horse, pig, and mouse lemur are described in Materials and Methods. GenBank accessions used for human, mouse, and rat genes are: **human** *KLRC1-KLRC3* (NM002259-NM002261), *KLRC4* (NM013431), *KLRD1* (AB009597), *KLRK1* (AF461811); **rat** *KLRC1* (NM001037441), *KLRC2* (NM019261), *KLRC3* (NM001029908), *KLRD1* (NM012745), *KLRE1* (NM181372), *KLRI1* (NM001012649), *KLRI2* (NM001012648), *KLRH1* (NM139187); **mouse** *KLRD1* (NM010654), *KLRE1* (NM153590), *KLRI1* (AY324874), *KLRI2* (NM177155), *KLRK1* (NM001083322), *KLRA2* (AF253058)


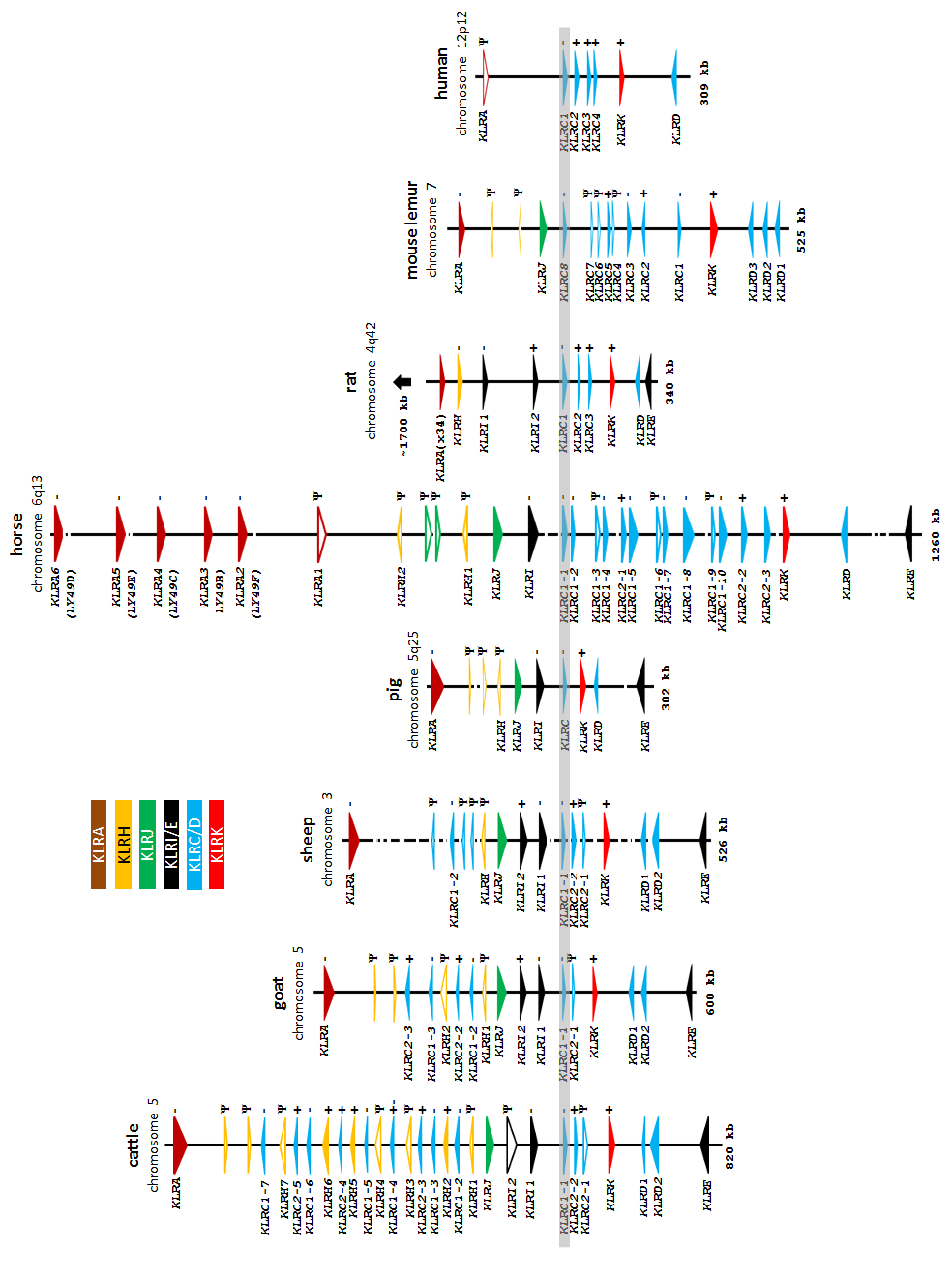


**Supplementary Fig. 2** Comparative organization of the NKC in selected genomes. Expanded from figure 2 to include the draft genome assemblies of the sheep, pig, and horse, and the BAC assembly of the mouse lemur NKC (GenBank: FP236838). Genomic regions are approximately to scale, visualized as in Fig. 2, and anchored on *KLRC1-1* (*shaded grey line*).

**Supplementary Table 1** Oligonucleotide sequences used for BAC screening

| Target gene | Orientation | Sequence (5’ – 3’) |
| --- | --- | --- |
| *KLRC1* | Forward | TGAGCAGGAATTAACATATGYWG |
| *KLRC1* | Reverse | CTTCTCTGRAGGTGAYGGTGAAC |
| *KLRC1* | Forward | GYAGAACWSAAGGSAGTCAA |
| *KLRC1* | Reverse | RSAGTSATGGGRRCARTTGK |
| *KLRD1* | Forward | GAGAATCCAAAAGGTTCTTTGGT |
| *KLRD1* | Reverse | CTCCACCTTGTGATGCTTATGTT |
| *KLRD2* | Forward | CTCCATTTCCTTCTCTACCAACA |
| *KLRD2* | Reverse | GCCTTTTGTTGATGGTAACTTTG |
| *KLRJ1* | Forward | AGGATCCGTACACACATACATTC |
| *KLRJ1* | Reverse | TGGGAAAATTATGACAATAGCAG |
| *GABARAPL1* | Forward | AATACCAAGGTGTCTTTCTGCAT |
| *GABARAPL1* | Reverse | CTTTTAAAGCAGTCATCCAAGGT |
| *STYK1* | Forward | GAAGAGAGTGGTGAGAAAGGAAT |
| *STYK1* | Reverse | CATCAGAGTCCTTGAGATTTGAC |

**Supplementary Table 2** *KLR* subgroups across species

| Gene | Alternative names | Human | Mouse lemur | Rat | Horse | Pig | Goat | Cattle |
| --- | --- | --- | --- | --- | --- | --- | --- | --- |
| *KLRA* | *Ly49* | (1)* | 1 | ~34 | 5(1) | 1 | 1 | 1 |
| *KLRH* | - | - | (2) | 1 | (2) | (3) | (4) | 3(6) |
| *KLRJ* | - | - | 1 | - | 1 | 1 | 1 | 1 |
| *KLRI* | - | - | - | 2 | 1 | 1 | 2 | 1(1) |
| *KLRC* | *NKG2A*,*C*,*E*,*F* | 3(1) | 4(3) | 3 | 10(3) | 1 | 5(1) | 11(1) |
| *KLRK* | *NKG2D* | 1 | 1 | 1 | 1 | 1 | 1 | 1 |
| *KLRD* | *CD94* | 1 | 3 | 1 | 1 | 1 | 2 | 2 |
| *KLRE* | - | - | - | 1 | 1 | 1 | 1 | 1 |

* Numbers in parentheses indicate pseudogenes and fragments
